# Growth of *Acinetobacter baumannii* in Pellicle Enhanced the Expression of Potential Virulence Factors

**DOI:** 10.1371/journal.pone.0026030

**Published:** 2011-10-27

**Authors:** Sara Marti, Yassine Nait Chabane, Stéphane Alexandre, Laurent Coquet, Jordi Vila, Thierry Jouenne, Emmanuelle Dé

**Affiliations:** 1 Laboratoire Polymères Biopolymères Surfaces, UMR 6270 & FR 3038 CNRS, University of Rouen, Mont Saint Aignan, France; 2 Department of Microbiology, Hospital Clinic, Barcelona, Spain; Columbia University, United States of America

## Abstract

**Background:**

Interestingly, *Acinetobacter baumannii* presents an enhanced capacity to form biofilms (also named pellicles) at the air-liquid interface as compared to the other *Acinetobacter* species. This characteristic questions the contribution of this phenotype to an increased risk of clinical infections by this pathogen.

**Methodology/Principal Findings:**

By a proteomic approach using 2-D gel electrophoresis-LC-MS/MS mass spectrometry, we compared the membrane protein patterns of *A. baumannii* 77, a pellicle-forming clinical isolate, grown in planktonic and in sessile modes. We identified 52 proteins with a differential expression, including 32 up-regulated and 20 down-regulated in the pellicle state. Several proteins, differentially expressed during pellicle development, were of particular interest. We determined the over-expression of four siderophore iron uptake systems including the acinetobactin and enterobactin receptors and confirmed that the development of this type of biofilm is promoted by ferric ions. Two over-expressed proteins, CarO and an OprD-homologue, putative carbapenem-resistance associated porins, would be involved in the transport of specific compounds, like ornithine, a biosynthesis precursor of a siderophore from the hydroxamate family. We evidenced the overexpression of a lipase and a transporter of LCFA that may be involved in the recycling of lipids inside the pellicle matrix. Finally, we demonstrated both by proteomic and by AFM studies that this particular type of biofilm required multiple pili systems to maintain this cohesive structure at the air-liquid interface; two of these systems have never been described in *A. baumannii*.

**Conclusions/Significance:**

Our study demonstrated that several proteins, overexpressed at a late state of pellicle development, could be potentially involved in virulence processes. Therefore, regarding the number of potential virulence factors that are over-expressed in this growth mode, the pellicle-forming clinical isolates should be kept under survey.

## Introduction

*Acinetobacter baumannii* is a nosocomial pathogen responsible of bloodstream and pulmonary infections that affects mainly critically ill patients in the intensive care units [1], [2]. Since the early 1980 s, this microorganism has emerged due to its extraordinary ability to adapt to the adverse hospital environment [1]–[3]. Nowadays, the global epidemiology is a cause of concern due to the widespread dissemination of this pathogen, which is commonly responsible of epidemic outbreaks in hospitals worldwide and may also persist for long periods of time, becoming endemic in certain hospitals. This persistence is mainly associated to a major resistance to antimicrobial drugs, together with the resistance to desiccation and disinfection [2]. The ability of *A. baumannii* to form biofilms that confer a protection from environmental hazards [4], [5] also contributes to these resistances.

A biofilm is a self-assembled microbial community, located in an interface and surrounded by a matrix of self-secreted polysaccharide material. This matrix acts as a protective layer and creates an optimal environment for genetic material exchange between the different microorganisms [6]. This bacterial mode of growth is an ancestral adaptation mechanism that allows bacteria to survive and colonize hostile environments [7]. Biofilm formation by nosocomial pathogens has been associated with some infectious diseases and device-related infections. It has been reported that biofilms might be responsible for the endemic occurrence and posterior epidemic outbreaks of *A. baumannii* in hospitals [6], [8]. Rodriguez-Baño *et al* [8] showed that biofilm-forming *A. baumannii* isolates were more susceptible to imipenem and ciprofloxacin than non biofilm-forming counterparts, which suggests that the survival of these isolates in the hospital environment was less dependent on antibiotic resistance.

Although biofilms may develop on a wide variety of interfaces, the best studied biofilms are the ones formed at the solid-liquid interface in which bacteria adhere to biotic or abiotic surfaces [9]–[11]. However, bacteria also form biofilms, generally named “pellicles”, at the air-liquid interface [4], [12]–[14]. This interface is a favourable niche for strictly aerobic bacteria which can obtain oxygen from the air and nutrients from the liquid media. Recently, we showed that *A. baumannii* and *Acinetobacter* genospecies 13TU, the two species responsible for most of the *Acinetobacter* infections [2], exhibit a higher ability to form pellicles than other less pathogenic species such as *Acinetobacter johnsonii, Acinetobacter lwoffii* and *Acinetobacter radioresistens* [15].

**Figure 1:**
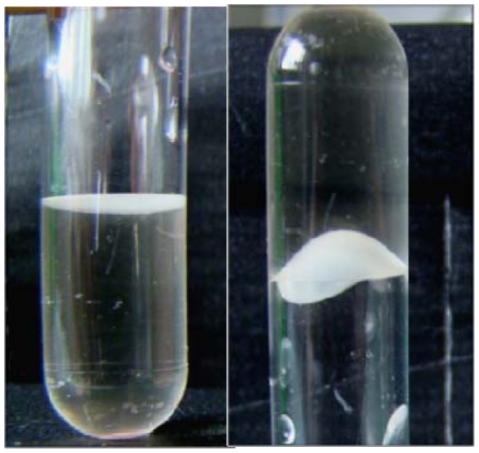
Pellicle formation by *Acinetobacter baumannii*. Left hand side: pellicle formation at the meniscus level; Right hand side: Turning the tube upside down shows evidence of pellicle cohesiveness and robustness.

These floating biofilms, which may be slightly attached at the meniscus to solid surfaces [13], form more complex structures and may require a higher level of organization due to the lack of a solid surface to initiate the attachment [16]. Furthermore, surface-associated bacteria present phenotypic changes with respect to gene transcription and growth rate when compared to free living bacteria; those modifications involve the expression of surface molecules, antibiotic resistance, virulence factors and nutrient utilization [6], [7]. In the present study, we characterized the membrane proteins associated to pellicle formation in an *A. baumanni* clinical isolate that was forming a very robust and physically cohesive pellicle (Figure 1). We compared the protein patterns of the cell envelope of this microorganism growing in planktonic and pellicle modes using a proteomic approach coupling two-dimensional gel electrophoresis and mass spectrometry. Our data suggest that the pellicle is a particular phenotype, during the formation of which, several potential virulence factors such as iron uptake systems or pili, are over-expressed.

## Materials and Methods

### Bacterial strain & growth conditions

*A. baumannii* strain 77 was a clinical isolate obtained from the Microbiology Department in the Hospital Clinic of Barcelona [17].

Bacterial growth was performed at 25°C in glass Erlenmeyers containing Mueller Hinton broth (MHB). For planktonic growth, bacteria were incubated to a final OD_600_ of 1.5 (stationary phase) under agitation. For the pellicle formation, the culture was performed for five days without shaking (Figure 1).

### Protein extraction

The pellicle was recovered with a 10 ml pipette from the surface of the culture, resuspended in 4 ml of Tris-NaCl Buffer (20 mM Tris-HCl/150 mM NaCl, pH 7.4) and broken down in an ultrasonic bath (Transsonic 950, Prolabo, Fontenay-sous-Bois, France): 10 s pulses for 3 min. Bacteria were harvested and resuspended in Tris-NaCl buffer.

For planktonic cultures, bacteria were harvested by centrifugation at 3,500×g for 30 min, washed and resuspended in Tris-NaCl buffer.

Protein extraction was performed for both samples, *i.e.* planktonic and pellicle cells as described by Marti *et al.* [18], with modifications. Briefly, cells were sonicated on ice for 5 min with intervals of 3 s and amplitude of 21% (Vibra Cell 75115, Bioblock Scientific, Illkirch, France). Unbroken cells were eliminated by centrifugation at 9,000×g for 10 min at 4°C. The supernatant was then centrifuged at 100,000×g for 45 min at 4°C using a Beckman Coulter TL100 ultracentrifuge. The pellet containing the cell envelope proteins (insoluble) was re-dissolved in distilled water.

### Two-dimensional gel electrophoresis (2-DE)

The analyses of the protein extracts were performed by 2-DE. For each extraction, 150 µg of proteins were added to IEF buffer (7 M urea, 2 M thiurea, 0.5% ampholytes pH3-10, 2 mM TBP, 1.5% ASB-14, 0.005% Bromophenol Blue) and solubilized for 60 min at room temperature with slight shaking. Samples were then submitted to 3 freeze-defrost steps and stored at −20°C overnight.

The first-dimensional gel separation was carried out using ReadyStrip™ IPG Strip (18 cm, pH 3-10 NL, Bio-Rad, Marnes-la-Coquette, France). After 24 h of passive rehydration of the strip with IEF buffer, the protein sample was added to the strips through a loading cup placed at 1.5 cm from the cathode. Isoelectric focusing (IEF) was performed with the Ettan IPGphor 3 System (GE Healthcare, Orsay, France) in four steps (31,500 Vh): 500 V for 1 h, 1,000 V gradient, 10,000 V gradient and 10,000 V for 2 h. After two equilibration steps with 2% DTT and 2.5% iodoacetamide respectively, the second dimension, i.e. a SDS-PAGE, was performed on a Protean IIXi cell (Bio-Rad) using a 10.5% (w/v) polyacrylamide resolving gel (width, 16 cm; length, 20 cm; thickness, 0.75 mm). After migration, proteins were visualized by a silver nitrate staining as described by Rabilloud *et al* [19].

### Gel Image acquisition and analysis

All gels were performed in duplicate from three independent protein extractions. Image acquisition was carried out on a ProXPRESS™ Proteomic Imaging System (PerkinElmer Inc), and image analysis was performed using the Progenesis SameSpots V2 software (Nonlinear Dynamics, Newcastle upon Tyne, UK) for gel alignment, expression, including principal component analysis and hierarchical clustering analysis. Protein spots from the two bacterial modes of growth were considered to display significant quantitative differences when the *p* value<0.05 (t-test), fold change >1.5 and a power value >0.8.

### Protein identification by LC-MS/MS

Excised spots were washed several times with water and dried for 2 hours. Trypsin digestion was performed overnight with a dedicated automated system (MultiPROBE II, PerkinElmer). The gel fragments were subsequently incubated twice for 15 min in a H_2_O/CH_3_CN solution to allow peptide extraction from the gel pieces. Peptide extracts were dried and dissolved in starting buffer for chromatographic elution, consisting of 3% CH_3_CN and 0.1% HCOOH in water. Peptides were enriched and separated using a lab-on-a-chip technology (Agilent, Massy, France) and fragmented using an on-line XCT mass spectrometer (Agilent). The fragmentation data were interpreted using the Data Analysis program (version 3.4, Bruker Daltonic, Billerica, MA, USA). For protein identification, MS/MS peak lists were extracted, converted into mgf-format files and compared with the protein database using the MASCOT Daemon (version 2.1.3; Matrix Science, London, UK) search engine. Searches were performed with no fixed modification and with variable modifications for oxidation of methionines, and with a maximum of one missed cleavage. MS/MS spectra were searched with a mass tolerance of 1.6 Da for precursor ions and 0.8 for MS/MS fragments. Only peptides matching an individual ion score >51 were initially considered. Proteins with two or more unique peptides matching the protein sequence were automatically considered as a positive identification.

### Iron effect on biofilm formation

The role of iron on biofilm formation was tested by using M9 minimal medium supplemented with 40 µM FeCl_3_ and/or 200 µM 2,2-dipyridyl (DIP) to simulate iron-depleted and iron-limited conditions. One milliliter of M9 medium was inoculated with an overnight culture at initial OD_600_ of 0.01 in polypropylene (13×75 mm) sterile tubes. Cultures were incubated for 24 h or 5 days without shaking at 25°C. The cells attached to the tube walls were visualized and quantified by staining with crystal violet as described by O'Toole and Kolter [20]. After rinsing tubes with water, attached cells were stained by incubation 20 min with 0.1% crystal violet. Crystal violet was solubilized by adding 1 ml of ethanol for each tube and the OD_570_ was finally measured. Data are presented as the mean ± standard deviation (SD). Assays were performed in triplicates. P values were obtained by a Student t-test using the GraphPad Prism software.

### Atomic Force Microscopy (AFM)

*A. baumannii* pellicles were transferred to a solid support after 24 h growth. Water-facing side pellicles were transferred by carefully landing collodion-coated glass slides on the pellicle surface and by removing the sample after contact; the slides were rinsed with milli-Q water and dried slowly in the air for 24 h. Air-facing side pellicles were transferred by dipping a muscovite slide in the liquid phase followed by a careful lifting up of the slide; these slides were then dried slowly in the air for 24 h. All samples were placed in a desiccator cabinet for at least 24 hours before AFM imaging.

For imaging *A. baumannii* planktonic bacteria, a few droplets of bacterial suspension were dropped on collodion-coated glass or on muscovite slides. After one minute, the samples were rinsed four times with milli-Q water. Samples were then dried in a desiccator cabinet for at least 24 hours before AFM imaging.

AFM imaging was performed by using a Nanoscope III Multimode microscope (Veeco instrument, Santa Barbara, Ca, USA) with a 100 µm piezoelectric scanner. Imaging was achieved in the air in the contact mode. The cantilevers used were characterized by a low spring constant of about 0.06 N/m and were equipped with sharpened Si-tips. All the measurements were performed with the feedback loop on (constant force from 10−9 to 10−8 N). All images are presented in the height mode (black and white palette for height: dark for low zones, light for high zones) and are topview images. A flatten operation was usually done on all images. This was achieved either with the Nanoscope software (flatten order 2 or 3) or using the free Gwyddion AFM software downloadable at http://gwyddion.net/ (three points levelling, polynomial background order 2).

Bacteria sizes and heights were made using the profile tool in Gwyddion. In order to minimize the noise, profiles were averaged from 10 adjacent profiles. Bacteria sizes were taken from 5×5 µm square images (from 4 images −20 bacteria- for the planktonic samples and from 5 images −50 bacteria- for the pellicle samples). Height measurements were made on 4 images −8 bacteria- and one image −2 bacteria- for respectively the collodion and mica deposited planktonic samples. Height measurements for the pellicle samples were taken from 2 images −15 bacteria- and 2 images −17 bacteria-for respectively the collodion and mica deposited planktonic samples. Pili lenghts were measured using the Gwyddion measuring tool and the results were compiled. A histogram was plotted and a gaussian fit was done. The average length was also calculated. Respectively 69 and 99 pili were used for planktonic and pellicle samples. All values are presented with a standard error using a confidence interval of 95%.

## Results

### 2-DE analysis and LC-MS/MS identification

**Figure 2:**
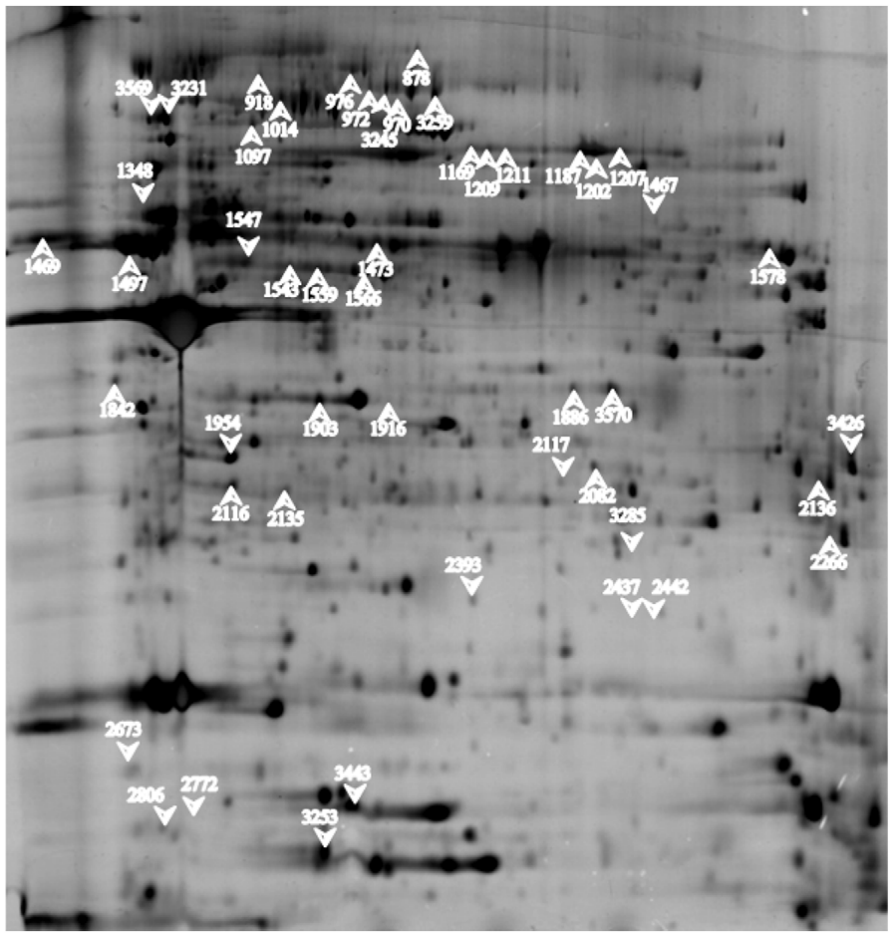
Representative 2D electrophoresis gel corresponding to the pellicle state of growth. Up-facing arrow: proteins over-expressed; Down-facing arrow: proteins under-expressed.

A total of 488 spots were matched across all the gels using SameSpots software analysis (a representative 2D gel image is shown in Figure 2). Ninety-four spots exhibited a significantly (*i.e.*, fulfilled the criteria used for comparative analyses) altered amount in planktonic or pellicle modes of growth. LC-MS/MS analyses successfully identified 52 proteins, including 32 up-regulated and 20 down-regulated in the pellicle state (Tables S1 & S2). The identification carried out using the Mascot Daemon search engine gave results from different *A. baumannii* genomes; therefore, we considered necessary to homogenize all these results in order to be able to compare them. For this purpose, we used *A. baumannii* strain AYE [21] as reference strain (Tables S1 & S2). For gene homology, we used the data from the AcinetoScope project realised by the Genoscope (French National Sequencing Centre), which offers the full genome sequence of several *A. baumannii* strains, together with the synteny maps showing the similarity in gene function between the different bacterial genomes (www.genoscope.cns.fr).

From the 52 identified proteins, we designed different functional classes: 1] Porins (8 proteins, 3 down-regulated and 5 up-regulated in pellicle state); 2] Inorganic ion transport (12 proteins, 4 down-regulated and 8 up-regulated); 3] Cellular metabolism (11 proteins, 8 down-regulated and 3 up-regulated); 4] Starvation (2 down-regulated proteins); 5] Hypothetical proteins (3 up-regulated proteins); 6] Bacterial pili (8 up-regulated proteins); 7] Lipid biosynthesis (3 down-regulated proteins); 8] Lipid and carbohydrate transport (5 up-regulated proteins).

Several spots (2393, 2437 and 2442) were identified as containing the same protein, *e.g.*, the structural outer membrane protein OmpA.

### Effect of iron-limited growth conditions on pellicle formation

**Figure 3:**
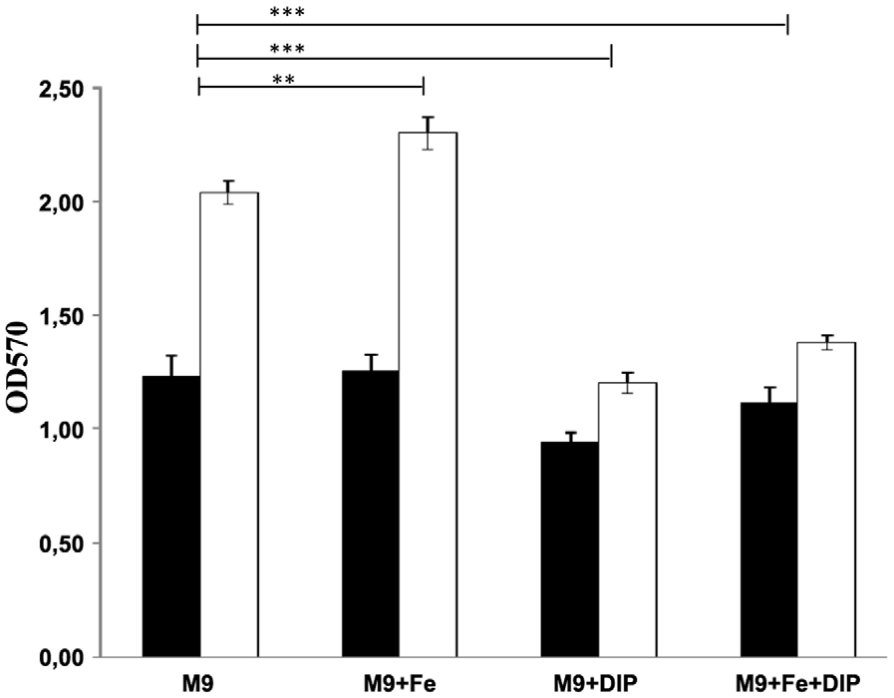
Iron effect on biofilm formation. Cells were cultured at 25°C in polypropylene tubes in M9 minimal medium with no supplementation or supplemented with 40 µM FeCl_3_ or 200 µM DIP or with 40 µM FeCl_3_ and 200 µM DIP for 24 h (▪) or 5 days(□). OD570 was determined after the crystal violet staining. P<0.001: ***; P<0.05: **.

The over-expression of iron-uptake systems by cells in 5-days old pellicles leads us to examine the impact of the presence of ferric ion in the growth medium. As shown in the figure 3, the addition of inorganic iron (40 µM FeCl_3_) to M9 minimal medium increases the amount of attached cells, more significantly for 5 days than for 24 h, as compared to iron-free M9. In contrast, the supplementation of the medium with the iron chelator DIP or with both, iron plus DIP, decreased cell attachment to polypropylene significantly, showing thus the significance of this compound for this growth mode.

### Atomic Force Microscopy

Planktonic *A. baumannii* cells were imaged as reference. When deposited on muscovite, cells were quite fragile and difficult to image by AFM either in the contact or tapping mode. When deposited on collodion-coated glass slides, they appeared as individual, twins or small aggregates of a few bacteria and could be easily imaged. The single cells were egg-shaped with a ratio length/width of 1.1; the average length was 990±60 nm and the width 870±70 nm. The average height of the bacteria was measured as 275±20 nm on collodion-coated glass slides and 320±20 nm on muscovite slides. These differences may be due to slide nature: the collodion-coated glass slide presented a rough soft polymer surface whereas the muscovite slide exhibited a flat mineral surface.

**Figure 4:**
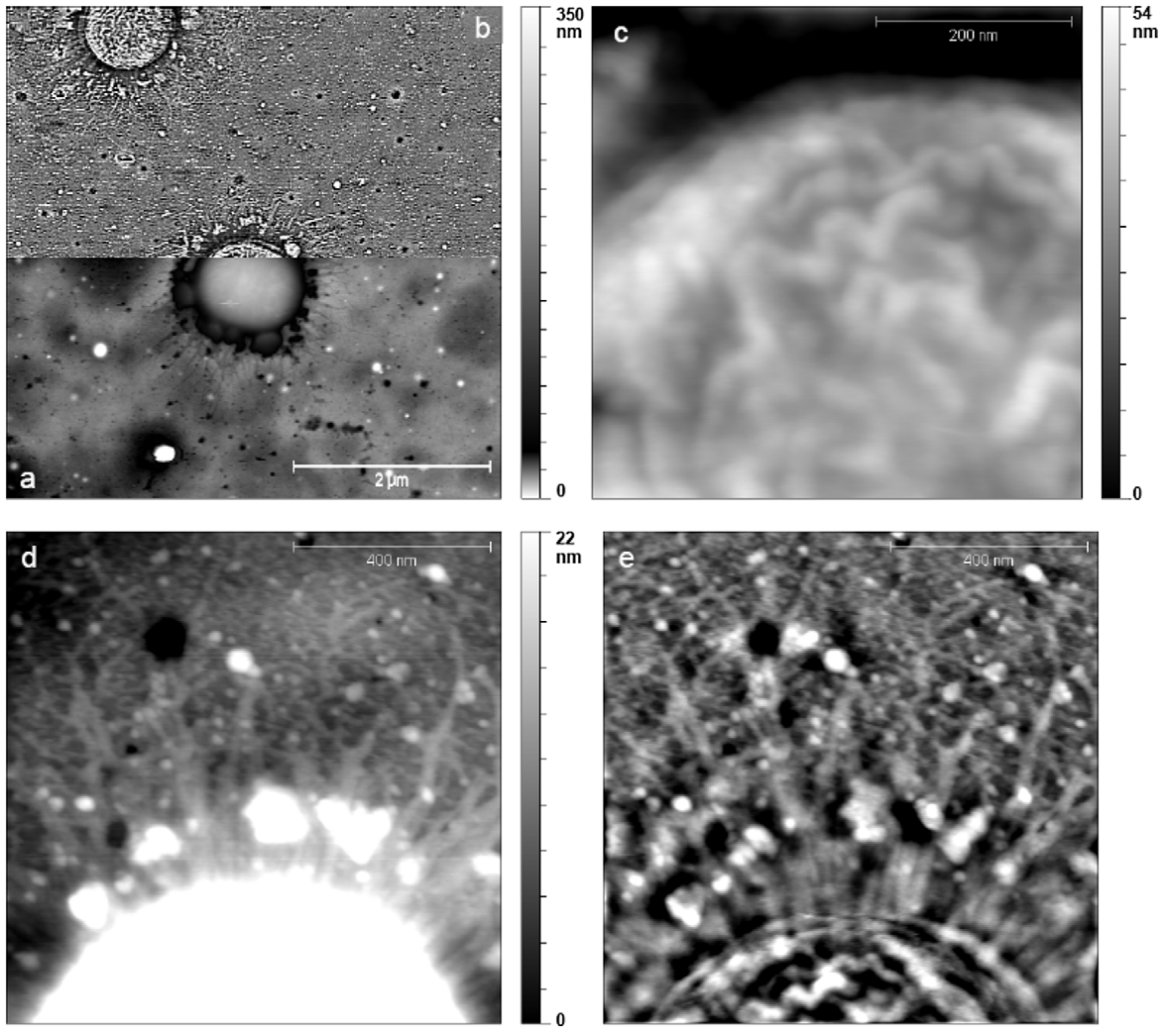
AFM images of an *A. baumannii* bacterium grown in solution. Deposition on a collodion-covered glass slide. a) + b) image 5 µm×5 µm – the bottom part of the image (**a**) is the classical topographic representation using an uncommon scale made to emphasize the pili while the top part of the image (**b**) is obtained after a high-pass filtering in order to emphasize the ripple structure of the bacteria as well as the pili. **c**) zoom image 500 nm×500 nm showing the ripple structure of the bacterial envelope. **d**) and e) zoom image 1 µm×1 µm showing the pili. **e**) obtained after a Fourier transform filtering allowing the observation of the small radial pili.

An isolated *A. baumannii* bacterium is shown on figure 4 a & b. Pili, ranging from 0.3 to 1 µm length, were spread all around the bacterium. The average length of these pili were found to be 0.6±0.3 µm. Zooming on the bacterial surface (Figure 4 c) clearly exposed the ripple structure of the bacterial envelope already seen on the image obtained after performing a high-pass filter (Figure 4 b). Zooming on the edge of the bacterium revealed the presence of radiant linear small pili with an estimated length of about 100 nm (Figure 4 d and e). The diameter of these pili was estimated to range from 10 to 20 nm, clearly showing that they were not individual pili but bundles [22].

**Figure 5:**
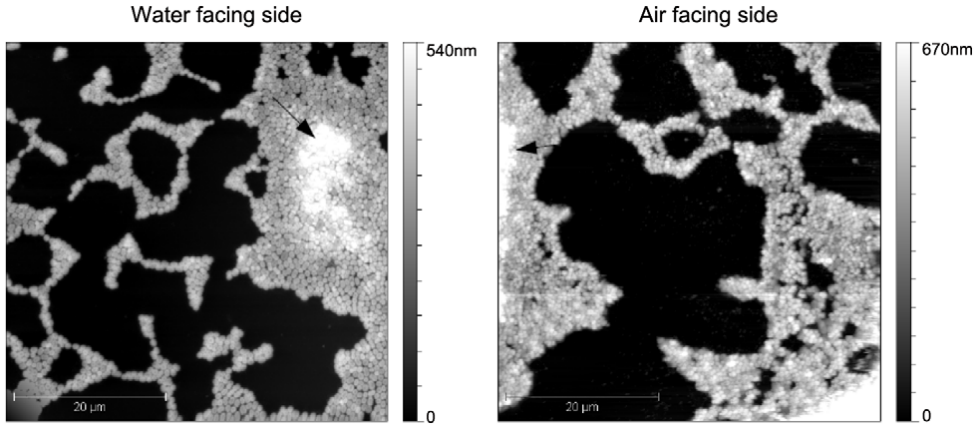
AFM images of the *A. baumannii* pellicles (50 µm×50 µm). Water-facing side (left) and air-facing side (right) in the classical topographic representation. Images are done on the edge of a large colony. The bright parts of the images correspond to a bacterial bilayer (see arrows).

The pellicles were examined by atomic force microscopy, comparing the structural and morphological characteristics present at both sides of this structure. Pellicles were imaged after a 24 h growth to allow colony border analysis. Images of the air-facing and water-facing sides of the pellicle were quite similar on a large scale (Figure 5). Fairly big colony islands of more than 100 µm diameter were observed with the presence of microcolonies at their vicinity. Within the bacterial pellicle, holes (500 nm maximum diameter) were also visible which could be useful for the feeding of the bacteria with organic matter as well as oxygen [23]. The edge of the colonies was constituted of a bacterial monolayer (up to a distance of 7 µm), with a second bacterial layer covering the colony central area (see arrows in Fig. 4). Three-dimensional bacterial clusters could be built within big colonies. The maximum height we were able to measure was around 5 µm. The size of the bacteria in both, air-facing or water-facing, side pellicles was about 850±70 nm in length and 740±70 µm in width. The length/width ratio was about 1.1. The average height of a bacterium was 390±30 nm on the water-facing side pellicle while it was 500±50 nm on the air-facing side pellicle. Once again these differences in height may originate from differences in the nature of the slides. The height of the bacteria in biofilm was higher than that of the planktonic bacterium while the length and width were slightly smaller.

**Figure 6:**
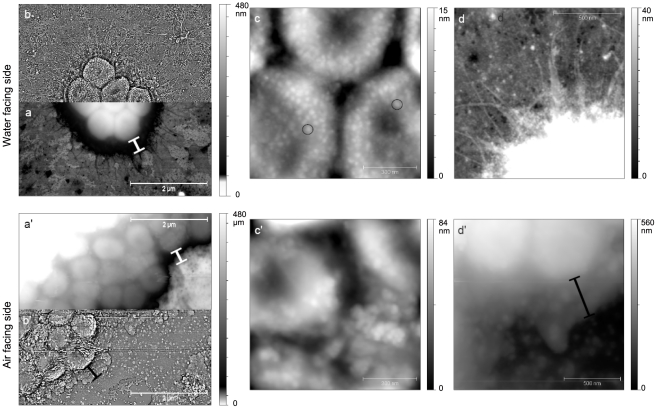
AFM of the *A. baumannii* pellicles at a lower scale. Water-facing side (top images) and air-facing side (bottom images). Image of the air-facing side pellicle is made at the border of a big colony. Image of the water-facing side pellicle is done on a small colony. a) + b) and a′) + b′) images 5 µm×5 µm – **a**) and **a′**) are the classical topographic representation using an uncommon scale, while **b**) and **b′**) are obtained after a high-pass filtering. The measurement marks indicate the EPS surrounding layer. **c**) and **c′**) zoom images 1 µm×1 µm show the differences in structure of bacterial envelope between the water-facing side bacteria (c) and the air-facing side (c′) The circle in c) marks some circular sub-structures (mean diameter of 3 nm). **d**) and **d′**) Images 1,25 µm×1,25 µm. d) clearly show the pili and d′) emphasize the surrounding EPS layer (see measurement mark).

Images at a lower scale showed more differences between both sides of this pellicle (Figures 5). The air-facing and the water-facing sides were surrounded by a layer (about 20 nm in height) which overflows the colony on a distance of about 500 nm and may be constituted by the EPS of the matrix (Figure 6c and 6c′ – see measurement marks)”.

Observation of the water-facing side pointed out the presence of the pili around bacteria, which were located on the border of the colonies (Figure 6 a, b and d). These pili ranged from 0.3 to 2,5 µm in length. The average length is 1.3±0,7 µm and is twice the average pili length in planktonic bacteria. When zooming on top of the bacteria, we observed circular sub-structures with a diameter of about 3 nm and an average height of 1 nm that may be the points of attachment of pili (Figure 6c, in circles). The disposition of those pili suggests that pellicle forming bacteria need those pili to float at the liquid/air interface.

With respect to the air-facing side of the pellicle, sub-structures were not observed on the top of the bacteria (Figure 6c′). Instead, we observed a rather rough surface (Figure 6d′), probably of the same nature than the surrounding layer, which was most likely the EPS secreted to protect bacteria from the environment. It should be noticed that we were not able to see pili on the air-facing side of the pellicle. This might be due to the pili being compacted during the transfer and covered by the surrounding EPS layer hiding them in our measurements.

## Discussion

Survival in the hospital environment is one of the most challenging tasks facing the microorganisms and they have developed adaptation mechanisms to tolerate these adverse conditions. The major factors contributing to the persistence of *A. baumannii* in the hospital environment are the high intrinsic resistance to antibiotics and disinfectants, together with the ability to resist to desiccation [2], [24], [25]. Probably, in the harsh environmental conditions encountered in hospitals, the capacity of the bacterium to form biofilms is a decisive advantage for its survival. We have recently demonstrated that *A. baumannii* and *Acinetobacter* genospecies 13TU, the species most frequently involved in *Acinetobacter* nosocomial infections, presented an enhanced and non anecdotic capacity to form pellicles when compared to the other *Acinetobacter* species [15]. Therefore, we considered interesting to identify the differential molecular mechanisms expressed in *A. baumanni* living in a pellicle state, as opposed to the free-floating counterparts. These mechanisms may play a role in the *A. baumannii* persistence and maybe virulence. As the cell envelope is the first bacterial compartment in contact with the extracellular environment, it plays a crucial role in the bacterial responses to environmental alterations and in the virulence response [1], [26]–[30]. This leads us to compare the envelope protein patterns for bacteria grown in the two modes of culture, *i.e.* in suspension and in pellicle.

Although the protocol for protein extraction was the same for both protein extracts, one of their main differences was the high number (14 proteins) of contaminant cytoplasmic proteins overexpressed in samples extracted from planktonic cells. This cytoplasmic contamination is not surprising and it can reach nearly 30% of the overall membrane proteins whatever the extraction process used [31], [32]; nevertheless, the amount of overexpressed cytoplasmic proteins identified in the sample from the pellicle is relatively low if we compare it with the planktonic state (12.5% versus 65%, respectively). We noticed that, with the exception of the putative tRNA/rRNA methyltransferase (spot 2117), all the proteins associated to transcription and translation processes, as well as the putative stress protein and the SucB succinyltransferase protein, overexpressed in planktonic growth, have already been described as cytoplasmic contaminants in 2D and 1D membrane protein analysis [9], [32], [33].

The overexpression of all these cytoplasmic proteins (metabolic and lipid biosynthesis) would confirm a higher cellular activity in planktonic growth mode when compared to pellicle growth [34]–[36]. Surprisingly, stress proteins like starvation-associated proteins, generally observed in sessile bacteria [11], [37], were only overexpressed by planktonic microorganisms, suggesting differences between bacteria grown in pellicle and in biofilms at the solid-liquid interface. However, these results also showed that pellicle cells were not merely stationary phase cells, confirming here previous data on biofilms that developed at the solid-liquid interface [37].

Membrane proteins, which were overexpressed in the pellicle, might be classified into 4 classes: iron uptake systems, porins, proteins involved in lipid transport/metabolism and bacterial pili.

### Iron uptake systems

Iron concentration in the surrounding media acts as an important environmental signal which will induce the production of adhesion factors and plays also a critical role in biofilm formation [38], [39]: free iron concentration should usually be kept between 1 and 100 µmol/L to promote biofilm formation [40]–[42]. However, it was shown for *A. baumannii* biofilms that addition of iron chelators promotes the formation of biofilms when growing on polystyrene [5]. Iron seems therefore a non-required compound for the growth of the biofilm at the solid-liquid interface. In agreement with these data, Shin *et al.* [43] have recently analysed the differences in outer membrane protein (OMP) expression between planktonic bacteria and those attached to a solid surface. They identified a single outer membrane iron receptor protein which was not a siderophore receptor. In our study, we observed an under-expression of bacterioferritins in the pellicle state of growth (spots 2772, 2806), which were replaced by outer membrane iron receptor proteins and more specifically by ferric siderophore receptors (spots 970, 976, 3245, 1956, 3570). Bacterioferritins are haem-containing proteins which have an iron-storing function in the cytoplasm due to their molecular architecture [44]. On the other hand, ferric siderophores are extracellular ferric chelators synthesised and secreted by bacteria as a response to iron restriction; these iron carriers actively acquire the iron from the surrounding media and transport it into the bacteria after solubilisation [44]. In the pellicle state, we identified the over-expression of two different ferric siderophore receptors (spots 970/3245, 976), together with FepA, a receptor of ferric enterobactin and colicins (spot 1014) and BauB, a putative ferric acinetobactin binding protein (spots 1956/3570): the acinetobactin is a type of ferric siderophore which has already been identified in several *A. baumannii* clinical isolates [45]. This suggests the necessity for bacteria in pellicles to actively search for iron and is confirmed by the enhancement of the pellicle formation under iron supplemented growth conditions (Figure 3). This phenomenon differed from previously published data [5], [43] and may originate from two factors: *i*) the difference of interfaces air-liquid or solid-liquid that were colonised by the bacteria and *ii*) as demonstrated by Sauer *et al.* [10], it could be associated to the physiological changes experienced by the bacterium during the different stages of biofilm development. In our study, pellicle biofilms probably reached the maturation step II where protein profiles undergo the greatest modifications compared to planctonic cells or cells in 1 day-old biofilm [10]. In *Pseudomonas aeruginosa*, Sauer and colleagues showed indeed a maximal over-expression of the L-ornithine-5-monooxygenase involved in the pyoverdine biosynthesis in 6-days old biofilms [10].

### Porins

Other markers of iron starvation in pellicles could also be found in the differential expression of the second group of outer membrane proteins, *i.e.* the porins. Indeed, we found the OmpA protein under-expressed in pellicle state (in 3 mass isoforms, spots 2442, 2437, 2393 – Table S1). This observation is in concordance with previous studies reporting the under-expression in attached cells of the *Yersinia ruckeri* OmpA (six isoforms), the *E. coli* OmpA and the OmpA homolog in *P. aeruginosa*, the porin OprF, [9], [33], [46]. It therefore points out similarities between biofilms formed at both solid-liquid and air-liquid interfaces, but it also confirms that sessile cells encountered conditions of iron starvation. Nguwo *et al.* [47] deciphering the iron response in *A. baumannii*, showed that the production of OmpA decreased under Fe-chelated conditions and that this iron-induced porin may have a potential role in iron metabolism [47]. On the other hand, previous studies have determined that the expression of the structural OmpA porin favours the development of robust biofilms on different hydrophobic surfaces [48]–[50]. Ma *et al.* [50] have also demonstrated that *E. coli* OmpA may influence the biofilm formation *via* an activation of the Cpx stress response system, the expression of which is induced during the initial adherence of *E. coli* to abiotic surfaces [51]. Hence considering these results and the role that this adhesin may play in the interaction of cells with abiotic surfaces [52], [53], an attractive hypothesis is that OmpA could act in the initiation step of the biofilm formation and the subsequent iron starvation conditions encountered in the maturation step would decrease its expression.

This under-expression of OmpA was complemented by an over-expression of three additional porins, *i.e.*, CarO (spots 2116, 2135), OprD homologue (spots 1543, 1559, 1566) and OprC (spot 878), the former being two carbapenem-resistance associated porins [54]–[56]. Indeed, both CarO and the OprD homologue were suggested to allow the selective uptake of basic amino acids (more precisely ornithine for CarO) and, by structural homologies, the uptake of imipenem [54], [56]. Several reports have already suggested a close relationship between the use of carbapenems and biofilm formation by *A. baumannii* clinical isolates [8], [39], although none of them has directly associated the presence of these porins with the biofilm formation. In our context, this mechanism of specific channel over-expression suggests the necessity for the bacterium to acquire additional nutrients or small metabolites for pellicle development. Again, the overexpression of CarO could be induced by iron starvation as it was reported in *A. baumannii* in Fe-chelating conditions of growth [47]. This protein, involved in the uptake of ornithine (or other basic amino acid), could enhance the entry of a biosynthesis precursor of a siderophore from the hydroxamate family [47], [57]. The over-expression of the OprD homologue channel remains unclear as its substrate is still unknown. It could be as diverse as the substrates of the 19 protein members of OprD family in *P. aeruginosa* [58]. Finally, we noticed the over-expression of the OprC (ABAYE3703), a porin that presents 49% of identity with the *P. aeruginosa* OprC. In this microorganism, the production of this copper-regulated channel was shown to be enhanced by anaerobiosis as well as another porin from the OprD family, *i.e.* the OprE channel [59]. Many reports underlined the oxygen gradient concentration existing in biofilms [35], [60]–[62] and our results question about the oxygen-limited conditions (microanaerobiosis) of growth within the pellicle that could explain the increase expression of both OprC and OprD homologue (that may be an OprE homologue) channels in *A. baumannii*.

### Proteins involved in lipid transport/metabolism

In *A. baumannii* pellicles, as in most biofilms, the protective matrix is made of extracellular polymeric substances (EPS), where the main component is the exopolysaccharide (52–86%, S. Marti, personal communication). However, proteins, especially extracellular enzymes, and lipids may also represent 1–40% of this matrix [63], [64]. Owing to the presence of these enzymes, the matrix is considered as an external digestive system degrading EPS components that can be recycled and utilized as carbon and energy sources [64]. In our study, some protein expression differences in pellicle state of growth could be directly associated to the degradation and transport of lipids. Indeed, the proteins over-expressed in the planktonic state were mainly cytoplasmic enzymes associated to lipid biosynthesis (spots 2081, 2640, 2837); on the other hand, in the pellicle sample, we identified a secretory lipase over-expressed as well as a FadL-like transporter (spots 1469, 1678). Proteins of the FadL family are, to date, the only known channels involved in the uptake and transport of small hydrophobic molecules, like long chain fatty acids (LCFA) [65]. The *E. coli* FadL protein, archetype of this family, allows the bacterium to grow on LCFA (>C_12_) and works in association with FadD that converts these fatty acids to acyl coenzyme A in the cytoplasm. Then, the fatty acid degradative genes of the β-oxidation cycle are de-repressed and LCFA are metabolized in acetyl-CoA to finally generate energy [66]–[68]. This type of transporter associated with expression of extracellular lipases or phospholipases (like LipA or PlcB) could allow *P. aeruginosa* to degrade phosphatydylethanolamine [67] or phosphatydylcholine [69] as one nutrient source, for example in the lungs of cystic fibrosis patients. In pellicles, this association may allow a recycling of phospholipids from the lysed cells, providing a supplementary energy source. Additionally, these FadL transporters have also been proposed to be involved in the first steps of bacterial adherence, leading to host colonisation {de Lima Pimenta, 2003 68/id} and phospholipases and lipases are well known virulence factors in *P. aeruginosa* as well as in *A. baumannii* [71], [72].

### Bacterial pili

*A. baumannii* is a non-motile bacterium able to form bacterial surface appendages, *i.e.* pili [2], [5], [73], [74]. In *A. baumannii* ATCC19606 grown on agar, two types of pili were described: short pili (5–140 nm) [74] and long extensions (140–1000 nm) [73]. Our AFM data obtained with planktonic-grown *A. baumannii* deposited on mica or collodion-covered glass slides showed similar types of pili with similar lengths (Figure 4). In pellicle, we identified different proteins associated to pili formation: two chaperone-usher system pili and a putative type III pilus. Tomaras *et al.* [5] have characterised the operon *csu*, a chaperone-usher pili assembly system that has been associated to biofilm formation on abiotic surfaces. Our results show that this type of pili is also present in the pellicle formed by *A. baumannii*; we have identified the chaperone protein (CsuC – spot 2136) and also the outer membrane usher protein (CsuD – spot 3259). Although the spot 3259 was initially identified as PapC, the homogenization to the *A. baumannii* strain AYE genome showed that this gene was adjacent to CsuD (ABAYE1323 & ABAYE1322 – Table S2) and they were independent from the Pap operon. Nevertheless, the P pilus which are encoded by the Pap operon were also over-expressed in the bacteria present within the pellicle; by homology with *A. baumannii* strain AYE, this type of pili is encoded in an operon which is different from *csu* (ABAYE1857 & ABAYE1858 – Table S2). P pili have been described in *E. coli* as virulence factors, associated to pyelonephritis, which mediate the attachment to kidney tissues [75]. Both pili (*csu* and P pilus) are assembled by the chaperone-usher pathway and we have identified both, the periplasmic chaperone and the outer membrane usher protein. In addition, a third type of pili (FilF) was also over-expressed in the pellicle. This is a rare type described as filamentous type III pili in *Burkholderia cepacia* cystic fibrosis non-epidemic clinical isolates [76]. AFM images (Figure 5) of water-facing side of pellicles allow the observation of long pili (0.5–2 µm) on the edges of colonies. However it was not possible to discriminate multiple surface appendages since the known dimensions of P-pili and CsuA/BABCDE-encoded extensions are to date similar [73], [77]. It should be noticed that small type pili would be anyway difficult to observe owing to the EPS layer surrounding the bacteria in pellicle (Figure 6). Although pili were present in both, planktonic and pellicle states (Figure 4 & 6), the difference in length between both conditions (100 nm and 300 to 1000 nm for planktonic vs 400 to 2500 nm for pellicle bacteria) corroborates the differences in protein expression.

To date, biofilm formation at the solid-liquid interface has been associated mainly to the *csu* pili [5]; however, we found that in the pellicle formed by *A. baumannii* at the air-liquid interface, different types of pili are required, not only to provide bacterial attachment but also to maintain the whole structure floating on the top of the liquid medium. This multiple expression of different pili systems required for bacterial adhesion could also contribute to the particular persistence of *A. baumannii* in hospital settings.

### Concluding Remarks

*A. baumannii* is usually considered as a low-virulence pathogen [1], [2] but the severity of the infections that it could cause is a challenging point. A recent DNA sequencing approach of *A. baumannii* 17978 identified a major pathogenicity island carrying genes homologous to the Legionella/Coxiella Type IV virulence/secretion apparatus, and other smaller islands with genes mainly involved in cell-envelope or pili biogenesis, iron uptake and lipid metabolism [53]. Several specific virulence determinants have been studied in more detail, like the expression of several iron acquisition systems [47], [78], [79], the involvement of phospholipases [71], [72] as well as the involvement of pili in adherence to host cells, first step of colonization and then of *A. baumannii* infection [73], [74]. Furthermore, the OmpA of *A. baumannii*, which could be delivered by outer membrane vesicles, was also identified as a potential virulence factor inducing host cell death [80], [81], and it was shown to be also required for attachment of *A. baumannii* to human alveolar epithelial cells [49]. This involvement of a porin in adhesion process to host cells or to fibronectine is not unique and was also demonstrated for OprE in *P. fluorescens* or OprQ & OprF in *P. aeruginosa* [82]–[84]. Therefore, the cell envelope is a provider of numerous proteins involved in virulence processes. Our study demonstrated that several proteins, overexpressed at a late state of pellicle development, could be potentially involved in virulence processes; we so identified *i*) four iron uptake systems, *ii*) three porins (one of them belonging to the same family as the OprE and OprQ proteins from *P. aeruginosa* [54], as well as *iii*) a lipase and transporter of LCFA that may be involved in the lipid metabolism [70], and *iv*) three systems of pili, two of which have never been described in *A. baumannii*. More than 35% of *A. baumannii* and 25% of *Acinetobacter* genospecies 13TU clinical isolates have the ability to form a pellicle at the air-water interface [15]. As demonstrated by our study, this particular phenotype should be kept under survey and studied for further virulence investigations.

## Supporting Information

Table S1 **Proteins over-expressed in the planktonic growth state.** *: OM-Outer Membrane; CM-Cytoplasmic Membrane; C-Cytoplasm; U-Unknown.
(DOC)

Table S2 **Proteins over-expressed in the pellicle.** *: OM-Outer Membrane; C-Cytoplasm; U-Unknown; PP-Periplasm; EC-Extracellular.
(DOC)
