# Supplementary material for: Growth of Acinetobacter baumannii in Pellicle Enhanced the Expression of Potential Virulence Factors
Source: PLoS One. 2011 Oct 27;6(10):e26030. doi: 10.1371/journal.pone.0026030 (PMC3203104; doi:10.1371/journal.pone.0026030)
Supplement: Table S1 — Proteins over-expressed in the planktonic growth state. *: OM-Outer Membrane; CM-Cytoplasmic Membrane; C-Cytoplasm; U-Unknown. (DOC) [file pone.0026030.s001.doc]

**Table S1.** Proteins over-expressed in the planktonic growth state.

| **Spot** | **Anova (p)** | **Fold** | **Protein** | **Organism** | **Accession Number** | **Mascot Score** | **pI** | **MW (Da)** | **Cover (%)** | **Peptides Matched (score>51)** | **Functional Category** | **ABAYE** | **PSORT*** |
| --- | --- | --- | --- | --- | --- | --- | --- | --- | --- | --- | --- | --- | --- |
| **Outer Membrane Porins** | | | |  |  |  |  |  |  |  |  |  |  |
| **2442** | 1.44E-06 | 7.7 | OmpA | *A.baumannii* ATCC 17978 | gi|126642864 | 207 | 5.13 | 37342 | 11 | 4(2) | Cell wall / membrane biogenesis | ABAYE0640 | OM (10) |
| **2437** | 2.52E-06 | 6.3 | OmpA | *A.baumannii* ATCC 17978 | gi|126642864 | 259 | 5.13 | 37342 | 16 | 5(3) | Cell wall / membrane biogenesis | ABAYE0640 | OM (10) |
| **2393** | 2.62E-04 | 2.4 | OmpA | *A.baumannii* ATCC 17978 | gi|126642864 | 258 | 5.13 | 37342 | 15 | 4(4) | Cell wall / membrane biogenesis | ABAYE0640 | OM (10) |
| **Inorganic Ion Transport** | | | | |  |  |  |  |  |  |  |  |  |
| **3285** | 0.005 | 1.9 | K^+^-transporting ATPase C chain | *A.baumannii* SDF | gi|169633263 | 267 | 6.05 | 23081 | 28 | 4(3) | Inorganic ion transport and metabolism | ABAYE1422 | CM (9.82) |
| **2772** | 3.98E-05 | 4.1 | Bacterioferritin | *A.baumannii* ATCC 17978 | gi|126640856 | 299 | 5.02 | 18023 | 49 | 6(4) | Inorganic ion transport and metabolism | ABAYE3009 | C (9.97) |
| **2806** | 6.82E-05 | 3.1 | Bacterioferritin | *A.baumannii* ATCC 17978 | gi|126640856 | 388 | 5.02 | 18023 | 47 | 7(3) | Inorganic ion transport and metabolism | ABAYE3009 | C (9.97) |
| **3569** | 2.21E-04 | 1.8 | Outer membrane receptor for ferric coprogen and ferric-rhodotorulic acid | *A.baumannii* AB900 | gi|239501541 | 212 | 4.98 | 79613 | 8 | 4(3) | Inorganic ion transport and metabolism | ABAYE1494 | OM (10) |
| **Cellular Metabolism** | | | | |  |  |  |  |  |  |  |  |  |
| **1467** | 8.69E-04 | 3.1 | Transcription termination factor Rho | *A.baumannii* SDF | gi|169634328 | 528 | 7.02 | 47497 | 31 | 10(5) | Transcription | ABAYE3156 | C (9.97) |
| **3231** | 3.53E-04 | 1.7 | 30S ribosomal protein S1 | *A.baumannii* ATCC 17978 | gi|126641617 | 400 | 5.57 | 52602 | 22 | 8(5) | Translation | ABAYE2061 | C (9.97) |
| **3426** | 0.017 | 1.9 | 50S ribosomal protein L1 | *A.baumannii* AYE | gi|169797456 | 724 | 9.54 | 23842 | 69 | 13(8) | Translation | ABAYE3492 | C (9.26) |
| **3443** | 9.43E-04 | 1.7 | 50S ribosomal protein L9 | *A.baumannii* ATCC 17978 | gi|126642218 | 207 | 5.67 | 15771 | 28 | 3(2) | Translation | ABAYE1382 | C (9.97) |
| **1547** | 0.004 | 1.4 | Elongation factor Tu | *A.baumannii* ATCC 17978 | gi|162286746 | 467 | 4.99 | 41246 | 20 | 8(7) | Translation | ABAYE2946 | C (9.97) |
| **1954** | 9.70E-04 | 1.8 | Elongation factor Ts | *A.baumannii* ATCC 17978 | gi|126642362 | 910 | 5.27 | 30691 | 54 | 15(10) | Translation | ABAYE1154 | C (9.97) |
| **2117** | 4.20E-04 | 2.2 | Putative tRNA/rRNA methyltransferase | *A.baumannii* ATCC 17978 | gi|126640406 | 238 | 7.71 | 24314 | 34 | 5(3) | Translation | ABAYE3449 | C (9.26) |
| **1348** | 0.001 | 3.1 | SucB, dihydrolipoamide succinyltransferase | *A.baumannii* AYE | gi|169794947 | 263 | 5.23 | 42488 | 8 | 4(3) | Energy production and conversion | ABAYE0781 | C (9.97) |
| **Starvation** | | | |  |  |  |  |  |  |  |  |  |  |
| **2673** | 8.29E-04 | 1.9 | ClpXP protease specificity-enhancing factor | *A.baumannii* SDF | gi|169634297 | 213 | 4.77 | 15978 | 57 | 4(3) | General function prediction: Starvation response | ABAYE0485 | U |
| **3253** | 2.16E-04 | 1.7 | Putative stress protein | *A.baumannii* AYE | gi|169795610 | 347 | 5.62 | 15765 | 38 | 5(4) | Signal transduction mechanisms | ABAYE1500 | C (8.96) |
| **Lipid Biosynthesis** | | | | |  |  |  |  |  |  |  |  |  |
| **2081** | 5.86E-05 | 2 | UDP-N-acetylglucosamine | *A.baumannii* ATCC 17978 | gi|126642010 | 88 | 6.62 | 24767 | 15 | 2(1) | Lipid A biosynthesis | ABAYE1587 | C (9.97) |
| **2640** | 3.38E-04 | 3 | 3R-hydroxymyristoyl-ACP dehydratase | *A.baumannii* ATCC 17978 | gi|126642011 | 85 | 6.84 | 16335 | 17 | 2(1) | Lipid metabolism: Fatty acid biosynthesis | ABAYE1586 | C (9.26) |
| **2837** | 3.43E-04 | 3.4 | Biotin carboxyl carrier protein of acetyl-CoA carboxylase (BCCP) | *A.baumannii* ATCC 17978 | gi|126642055 | 82 | 4.81 | 13235 | 29 | 2(1) | Lipid metabolism: Fatty acid biosynthesis | ABAYE1538 | U |

*: OM-Outer Membrane; CM-Cytoplasmic Membrane; C-Cytoplasm; U-Unknown
