# Supplementary material for: Growth of Acinetobacter baumannii in Pellicle Enhanced the Expression of Potential Virulence Factors
Source: PLoS One. 2011 Oct 27;6(10):e26030. doi: 10.1371/journal.pone.0026030 (PMC3203104; doi:10.1371/journal.pone.0026030)
Supplement: Table S2 — Proteins over-expressed in the pellicle. *: OM-Outer Membrane; C-Cytoplasm; U-Unknown; PP-Periplasm; EC-Extracellular. (DOC) [file pone.0026030.s002.doc]

**Table S2.** Proteins over-expressed in the pellicle.

| **Spot** | **Anova (p)** | **Fold** | **Protein** | **Organism** | **Accession Number** | **Mascot Score** | **pI** | **MW (Da)** | **Cover (%)** | **Peptides Matched (score>51)** | **Functional Category** | **ABAYE** | **PSORT*** |
| --- | --- | --- | --- | --- | --- | --- | --- | --- | --- | --- | --- | --- | --- |
| **Outer Membrane Porins** | | | | |  |  |  |  |  |  |  |  |  |
| **2116** | 0.001 | 2 | Carbapenem-associated resistance protein precursor (CarO) | *A.baumannii* Ab242 | gi|83755431 | 128 | 4.8 | 26505 | 15 | 2(2) | Carbapenem resistance | ABAYE0924 | OM (9.52) |
| **2135** | 5.78E-05 | 2.8 | Carbapenem-associated resistance protein precursor (CarO) | *A.baumannii* Ab242 | gi|83755431 | 149 | 4.8 | 26505 | 15 | 2(2) | Carbapenem resistance | ABAYE0924 | OM (9.52) |
| **1543** | 2.16E-04 | 2.4 | OprD | *Acinetobacter* sp RUH2624 | gi|260551108 | 230 | 5.85 | 48126 | 18 | 6(2) | Multifunctional: putative transporter | ABAYE3674 | OM (9.93) |
| **1559** | 1.69E-05 | 2 | Putative outer membrane protein (OprD) | *A.baumannii* AYE | gi|169797605 | 707 | 6.09 | 47833 | 39 | 13(7) | Multifunctional: putative transporter | ABAYE3674 | OM (9.93) |
| **1566** | 4.67E-04 | 2 | Putative outer membrane protein (OprD) | *A.baumannii* AYE | gi|169797605 | 276 | 6.09 | 47833 | 16 | 6(2) | Multifunctional: putative transporter | ABAYE3674 | OM (9.93) |
| **Inorganic Ion Transport** | | | | |  |  |  |  |  |  |  |  |  |
| **1014** | 0.004 | 2 | Outer membrane receptor (FepA) | *A.baumannii* SDF | gi|169633929 | 265 | 5.75 | 82619 | 10 | 5(3) | Outer membrane receptor of ferric enterobactin and colicins B and D | ABAYE2812 | OM (10) |
| **1097** | 8.94E-06 | 1.9 | Outer membrane receptor protein, mostly Fe transport | *A.baumannii* AB900 | gi|239503323 | 502 | 5.42 | 78053 | 19 | 10(5) | Mostly Fe transport | ABAYE2648 | OM (10) |
| **878** | 1.67E-04 | 2.9 | Putative outer membrane copper receptor (OprC) | *A.baumannii* SDF | gi|169632179 | 582 | 5.78 | 77977 | 20 | 9(8) | Inorganic ion transport and metabolism | ABAYE3703 | OM (10) |
| **970** | 0.002 | 2.8 | Putative ferric siderophore receptor protein | *A.baumannii* ATCC17978 | gi|126640547 | 682 | 5.84 | 80006 | 28 | 10(7) | Inorganic ion transport and metabolism | ABAYE3290 | OM (9.95) |
| **976** | 6.29E-05 | 3.5 | Putative ferric siderophore receptor protein | *A.baumannii* ATCC17978 | gi|126641700 | 429 | 5.61 | 82325 | 16 | 8(5) | Inorganic ion transport and metabolism | ABAYE2001 | OM (10) |
| **3245** | 0.009 | 1.8 | Putative ferric siderophore receptor protein | *A.baumannii* ATCC17978 | gi|126640547 | 1232 | 5.84 | 80006 | 43 | 22(14) | Inorganic ion transport and metabolism | ABAYE3290 | OM (9.95) |
| **1956** | 3.72E-04 | 2.4 | Putative ferric acinetobactin binding protein (BauB) | *A.baumannii* AYE | gi|169795231 | 95 | 7.16 | 35945 | 7 | 2(1) | Inorganic ion transport and metabolism | ABAYE1092 | U |
| **3570** | 2.38E-05 | 2.5 | Putative ferric acinetobactin binding protein (BauB) | *A.baumannii* AYE | gi|169795231 | 230 | 7.16 | 35945 | 20 | 5(1) | Inorganic ion transport and metabolism | ABAYE1092 | U |
| **Bacterial Motility** | | | | |  |  |  |  |  |  |  |  |  |
| **918** | 2.09E-04 | 3.2 | P pilus assembly protein, porin PapC | *A.baumannii* ACICU | gi|184158131 | 214 | 5.43 | 94608 | 5 | 4(2) | Multifunctional: bacterial motility | ABAYE1858 | OM (10) |
| **3259** | 0.003 | 1.6 | P pilus assembly protein, porin PapC | *A.baumannii* ACICU | gi|184158735 | 1243 | 6.16 | 92666 | 34 | 23(14) | Multifunctional: bacterial motility | ABAYE1323 (CsuD) | OM (10) |
| **2266** | 6.73E-04 | 2.8 | P pilus assembly protein, chaperone PapD | *A.baumannii* ACICU | gi|184158132 | 476 | 9.44 | 27043 | 56 | 9(5) | Multifunctional: bacterial motility | ABAYE1857 | PP (10) |
| **2136** | 7.00E-04 | 2.8 | Protein CsuC ; putative type I pilus usher pathway chaperone | *A.baumannii* AYE | gi|169795448 | 513 | 9.34 | 30638 | 42 | 9(6) | Multifunctional: bacterial motility | ABAYE1322 | U |
| **1169** | 0.004 | 2.2 | Putative pilus assembly protein (FilF) | *A.baumannii* AYE | gi|169797112 | 256 | 6.82 | 69162 | 17 | 6(2) | Multifunctional: bacterial motility | ABAYE3123 | OM (9.49) |
| **1187** | 2.86E-04 | 3.1 | Putative pilus assembly protein (FilF) | *A.baumannii* AYE | gi|169797112 | 552 | 6.82 | 69162 | 19 | 9(7) | Multifunctional: bacterial motility | ABAYE3123 | OM (9.49) |
| **1202** | 2.22E-04 | 2.6 | Putative pilus assembly protein (FilF) | *A.baumannii* AYE | gi|169797112 | 515 | 6.82 | 69162 | 18 | 9(6) | Multifunctional: bacterial motility | ABAYE3123 | OM (9.49) |
| **1207** | 2.81E-04 | 2.7 | Putative pilus assembly protein (FilF) | *A.baumannii* SDF | gi|169634267 | 352 | 7.92 | 69222 | 15 | 8(2) | Multifunctional: bacterial motility | ABAYE3123 | OM (9.49) |
| **Hypothetical proteins** | | | | |  |  |  |  |  |  |  |  |  |
| **1903** | 0.002 | 2.6 | Hypothetical protein A1S_1462 | *A. baumannii* ATCC 17978 | gi|126641508 | 332 | 6.77 | 27178 | 28 | 6(2) | Putative Zn-dependent protease (TPR repeats) | ABAYE2192 | U |
| **1473** | 1.26E-04 | 1.7 | Hypothetical protein ABSDF0721 | *A. baumannii* SDF | gi|169632583 | 187 | 7.67 | 47029 | 11 | 3(2) | Cell wall/membrane biogenesis | ABAYE0730 | OM (9.52) |
| **2082** | 2.02E-04 | 1.9 | Putative signal peptide | *A. baumannii* ATCC 17978 | gi|126642168 | 323 | 6.97 | 29817 | 27 | 6(4) | Unknown function | ABAYE1438 | U |
| **Cellular Metabolism** | | | | |  |  |  |  |  |  |  |  |  |
| **1497** | 0.003 | 1.7 | Cell division protein FtsZ | *A. baumannii* AYE | gi|169794353 | 692 | 4.85 | 42039 | 46 | 12(8) | Cell cycle control, mitosis and meiosis | ABAYE0153 | C (9.12) |
| **1886** | 5.46E-04 | 1.6 | S-adenosylmethionine: 2-DMK methyltransferase and 2-octaprenyl-6-methoxy-14-benzoquinone methylase | *A. baumannii* ATCC 17978 | gi|126640428 | 694 | 6.45 | 34534 | 50 | 12(9) | Coenzyme transport and metabolism | ABAYE3424 | C (9.26) |
| **1916** | 0.003 | 1.8 | AraC family transcriptional regulator | *A. baumannii* ATCC 17978 | gi|126640495 | 152 | 5.67 | 29583 | 21 | 4(2) | Catalyzes formation of 5-phospho-alpha-D-ribose 1-phosphate | ABAYE3353 | C (9.97) |
| **Lipid and Carbohydrate Transport** | | | | |  |  |  |  |  |  |  |  |  |
| **1209** | 2.25E-06 | 4.1 | Putative Propiony-CoA carboxylase (Beta subunit) | *A. baumannii* ATCC 17978 | gi|126641421 | 200 | 5.87 | 55566 | 11 | 3(3) | Lipid transport and metabolism | ABAYE2289 | C (9.26) |
| **1469** | 4.21E-05 | 3.7 | Putative long-chain fatty acid transport protein | *A. baumannii* AYE | gi|169794882 | 76 | 5.12 | 50546 | 5 | 2(1) | Lipid transport and metabolism | ABAYE0711 | EC (9.65) |
| **1578** | 0.004 | 1.7 | Glucose/sorbone dehydrogenase | *A. baumannii* ACICU | gi|184158390 | 659 | 8.75 | 53116 | 35 | 12(8) | Carbohydrate transport and metabolism | ABAYE1605 | U |
| **1678** | 0.001 | 2 | Putative secretory lipase precursor | *A. baumannii* AYE | gi|169794306 | 96 | 6.79 | 43046 | 5 | 2(1) | Multifunctional | ABAYE0105 | PP (9.83) |
| **1842** | 0.002 | 1.9 | Putative lipoprotein | *A. baumannii* AB0057 | gi|213155752 | 357 | 4.87 | 38549 | 23 | 7(2) | Multifunctional | ABAYE3468 | OM (9.49) |

*: OM-Outer Membrane; CM-Cytoplasmic Membrane; C-Cytoplasm; U-Unknown
